# Adsorption and Kinetics Studies of Cr (VI) by Graphene Oxide and Reduced Graphene Oxide-Zinc Oxide Nanocomposite

**DOI:** 10.3390/molecules27217152

**Published:** 2022-10-22

**Authors:** Taiba Naseem, Fozia Bibi, Saira Arif, Muhammad Waseem, Sirajul Haq, Mohamad Nor Azra, Taavi Liblik, Ivar Zekker

**Affiliations:** 1 School of Engineering, Macquarie University, Sydney 2109, Australia; 2 Department of Chemistry, COMSATS University Islamabad, Islamabad 46000, Pakistan; 3 Department of Chemistry, University of Azad Jammu and Kashmir, Muzaffarabad 13100, Pakistan; 4 Institute of Marine Biotechnology (IMB), Universiti Malaysia Terengganu (UMT), Kuala Terengganu 21030, Malaysia; 5 Department of Marine Systems, Tallinn University of Technology, 19086 Tallinn, Estonia; 6 Institute of Chemistry, University of Tartu, Ravila 14a, 50411 Tartu, Estonia

**Keywords:** adsorption, chromium VI, graphene oxide, nanocomposites

## Abstract

In this work, graphene oxide (GO) and its reduced graphene oxide-zinc oxide nanocomposite (rGO-ZnO) was used for the removal of Cr (VI) from aqueous medium. By employing a variety of characterization techniques, morphological and structural properties of the adsorbents were determined. The adsorption study was done by varying concentration, temperature, pH, time, and amount of adsorbent. The results obtained confirmed that rGO-ZnO is a more economical and promising adsorbent for removing Cr (VI) as compared to GO. Kinetic study was also performed, which suggested that sorption of Cr (VI) follows the pseudo-first-order model. For equilibrium study, non-linear Langmuir was found a better fitted model than its linearized form. The maximum adsorption capacity calculated for GO and rGO-ZnO nanocomposite were 19.49 mg/g and 25.45 mg/g, respectively. Endothermic and spontaneous nature of adsorption was detected with positive values of ΔS (change in entropy), which reflects the structural changes happening at the liquid/solid interface.

## 1. Introduction

Industrial and agricultural pollutants alter the chemical, physical, and biological features of clean water [1]. Wastewater also contains elevated levels of micro-organisms such as protozoa, bacteria, and viruses, as well as poisonous radionuclides and trace elements. These effluents cause fatal waterborne diseases such as typhoid fever and cholera. Furthermore, long-term exposure of nickel (Ni), copper (Cu), chromium (Cr), silver (Ag), manganese (Mn), lead (Pb), and cadmium (Cd) can harm the human liver, kidney, brain function, and the nervous system, or even lead to death [2]. According to a report, every year, 72,000 children under-five die worldwide due to different diseases linked to unsafe water [3]. Various methodologies have been adopted for wastewater treatment, such as chemical filtration [4,5], water treatment [6,7], adsorption [8,9,10], and ion exchange [11]. Commercial metal oxide nanoparticles, Fe_2_O_3_, SiO_2_, NiO, and ZnO, were applied for water treatment [12]. It has been noted in literature that metal oxide nanoparticles’ behavior in water relies not only on their physical characteristics but also on their interactions with other water elements.

Methods to remove Cr(VI) from wastewater includes filtration membranes, reverse osmosis, electrochemical treatment, ion exchange, precipitation, adsorption/biosorption, and solvent extraction [13,14,15]. Among these, adsorption has seen widespread use due to its numerous advantages, including its high efficiency, low cost, and ease of operation. Carbon nanotubes (CNTs), GO, and fullerenes and their derivatives have shown high adsorption capacity as compared to traditional adsorbents such as silica, alumina, synthetic resins, and others. GO showed significant removal ability in the recovery of toxic heavy metal ions (As(III), Cd(II), Pd(II), Hg(II), and Cr(II)) [16]. In the literature, successful uptake of Cu (II), Ni(II), and Cd(II) from GO/PVA films was reported where equilibrium was achieved in a short time, and reusability of the membrane was observed six times [17]. The removal of Cr(VI) with various adsorbents has been recently studied [17,18,19,20,21]. Most of these adsorbents are limited in use because of low compatibility with natural water pH, poor adsorption ability, and prolonged contact time.

This paper focuses on a comparative study for the adsorption of Cr (VI) by GO and rGO-ZnO nanocomposite. For this purpose, an economic and simple method was used to prepare nanosized GO and rGO-ZnO. The reason for the selection of ZnO and its composite with rGO was its better performance for heavy metal ion removal [22,23,24]. The present study proved the rGO-ZnO nanocomposite as better adsorbent for Cr (VI) with excellent adsorption capacity as compared to GO. Various parametric studies have also been performed to determine the optimum parameter factors. It was deduced that the ideal pH value was 3 for the uptake of Cr (VI) [18]. ZnO particles possess a high surface area that is reasonable for adsorbing positive metal ions effectively [25,26].

## 2. Results

### 2.1. Characterization

After successful synthesis of nanocomposite, morphology and functional groups studies were performed by using various analytical techniques, including XRD, SEM, UV-Vis, and FTIR spectroscopies.

#### 2.1.1. X-ray Diffraction

**Figure 1:**
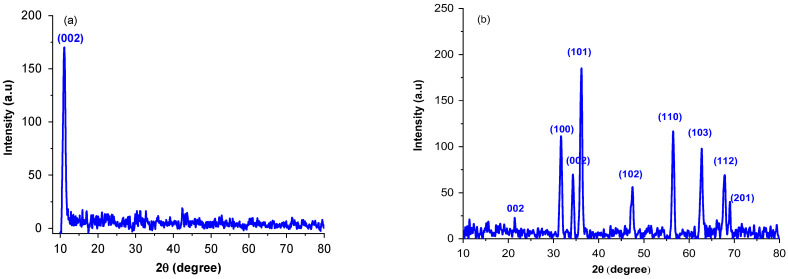
X-rays diffractograms for GO (**a**) and rGO-ZnO (**b**).

The XRD pattern are illustrated in Figure 1. The characteristic peak (002) of GO at 10.92° confirms the successful crystal formation for GO sheet and introduction of oxygenated functional groups into carbon sheet. For rGO-ZnO nanocomposite, the 2θ values were observed at 21.54°, 31.48°, 34.21°, 36.08°, 47.54°, 56.53°, 62.78°, 67.92°, and 69.36° corresponding to (002), (100), (101), (002), (102), (110), (103), (112), and (201) planes, respectively. These diffractions are associated to the hexagonal structure similar to the wurtzite (JCPDS 79-2205) [27]. The absence of diffraction peak at 10.92° in GO-ZnO infers that GO is reduced to rGO after hydrothermal treatment. Scherer’s formula (Equation (1)) was used to calculate the particle size:

(1) $$D=\frac{0.89\lambda }{\beta cos\theta }$$

where β = full width half maximum and θ = Bragg angle. The D_avg_ of rGO-ZnO was found to be 28 nm, which is slightly smaller than that reported in the literature [5]. For pure ZnO, it was 58 nm and decreased when it interacted with GO nanosheets. A small diffraction peak can be seen at 2*θ* = 21.54° (Figure 1b) that corresponds to rGO and it shows that rGO is completely dispersed and exfoliated into ZnO [23].

#### 2.1.2. Scanning Electron Microscopy

**Figure 2:**
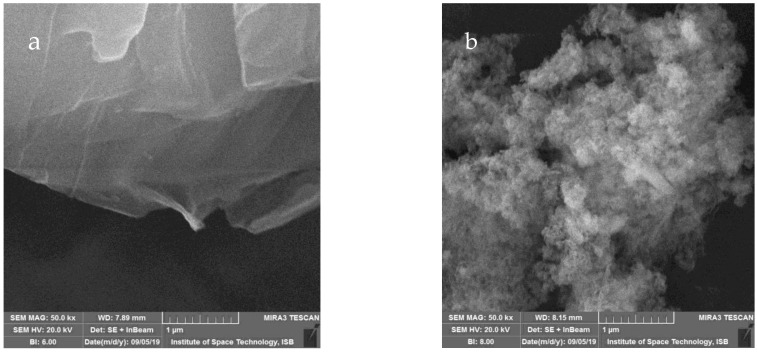
SEM images of GO (**a**) and rGO-ZnO (**b**).

The surface topography of synthesized samples was analyzed by SEM. Figure 2a depicts the surface morphology of GO, which shows that it is made up of many crumpled and stacked flakes with cavities which are closely connected. Edges of GO sheets tend to fold to form multilayers, which shows that a few layers of GO sheets can be prepared. There are also numerous wrinkles across the entire GO layer due to its crimping tendency. Figure 2b reveals that the nanocomposite of rGO-ZnO is in the form of aggregates and its average crystallite size is 30 ± 2 nm, which is in agreement with those reported in the literature [23]. It was noticed that on the GO surface, there is a homogenous rGO-ZnO nanocomposite deposition, which is due to the good coupling nature of ZnO.

#### 2.1.3. Fourier Transform Infrared Spectroscopy

**Figure 3:**
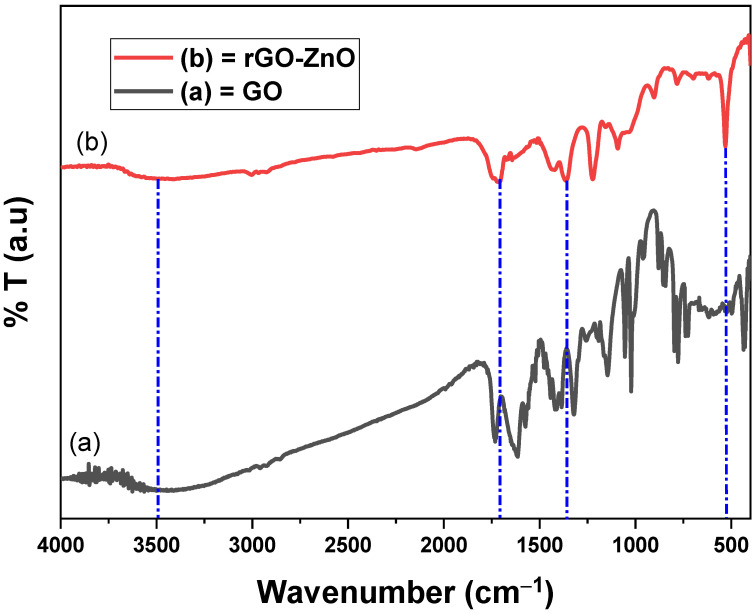
The FT-IR spectra of GO and rGO-ZnO.

The FT-IR of GO (Figure 3a) shows a broad and short absorption band at 3401 cm^−1^, which confirmed that O-H groups were retained on the surface of GO nanosheet [28]. The C-OH band was complemented at 1142 cm^−1^ due to O-H of GO. The bands at 1611 cm^−1^ and 1015 cm^−1^ correspond to the C=C of aromatic rings and vibrational stretch of C-O-C of alkoxy/epoxy. The majority of GO bands were found close to the reported values [29].

The OH group in the spectrum (Figure 3b) of rGO-ZnO was detected at 3995 cm^−1^ and it was found of almost the same intensity as in GO. The C-O stretching vibration was due to the absorption band at 1350 cm^−1^ of rGO-ZnO. The C=O stretching was due to band at 1701 cm^−1^. Zn-O vibrations were noted at 530 cm^−1^. The interaction of rGO with ZnO lead a significant reduction in OH band intensity, inferring that ZnO particles were effectively anchored on GO sheets [30].

#### 2.1.4. UV-Vis Spectroscopy

**Figure 4:**
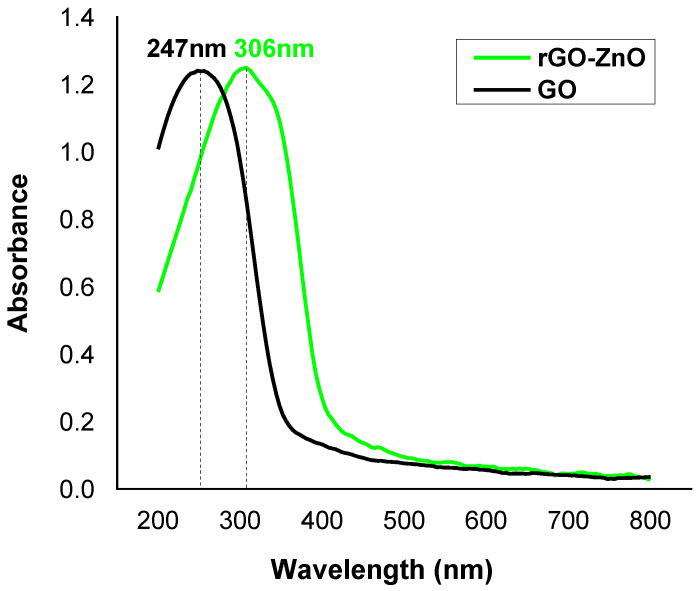
The UV-Vis spectral study of adsorbents.

The UV-Vis analysis was performed to find absorption band and band gap energy of nanocomposite. The UV spectrum (Figure 4) shows a peak at 247 nm, which is attributed to the π-π* transition in GO. For rGO-ZnO, it was found at 306 nm and is associated to n-π* transition in aromatic ring. The peak shifting to higher wavelength confirms a band gap narrowing of ZnO nanoparticles due to exfoliation of rGO into ZnO matrix, which is an indication of the formation of rGO-ZnO nanocomposite [31].

### 2.2. Water Purification by Adsorption

To assess sorption capability of rGO-ZnO, the effects of several operating parameters such as initial concentration, adsorbent dosage, pH, contact time, and temperature were explored for the removal of Cr (VI).

#### 2.2.1. Effect of the Initial Concentration

**Figure 5:**
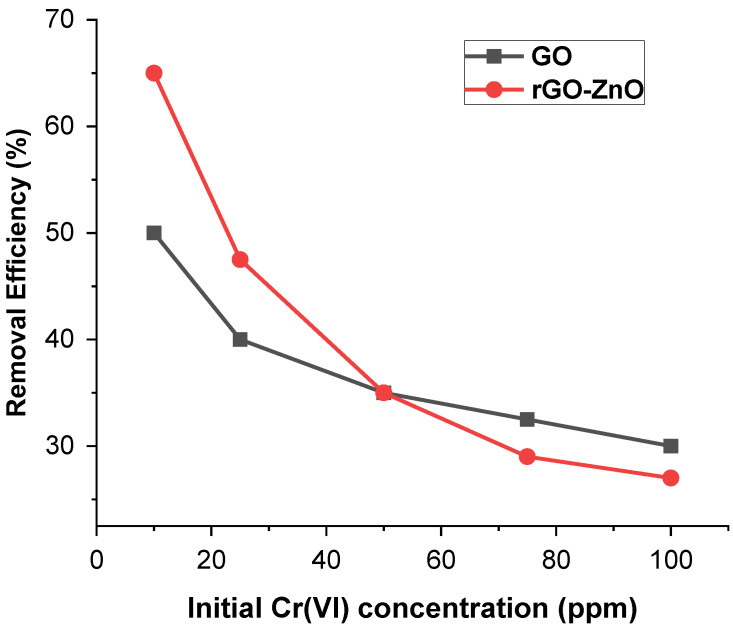
Effect of Cr (VI) concentration on its sorption.

To study the concentration effect, 60 mg of each adsorbent was added to 40 mL of (10, 25, 50, 75, and 100 ppm) solution at pH 3 keeping the other parameters constant. Figure 5 shows that the Cr(VI) removal trend of adsorbent decreases with increased metal ion concentration. At a lower concentration, the active sites to adsorbate ratio is high, and therefore, Cr (VI) ions efficiently interact with the sorbent, resulting in enhanced removal efficiency. These results indicate that by increasing the amount of adsorbate, removal efficiency decreased from 66.7% to 27.5% in the case of rGO-ZnO, whereas for GO, it was reduced from 49.8% to 30%.

#### 2.2.2. Effect of pH

**Figure 6:**
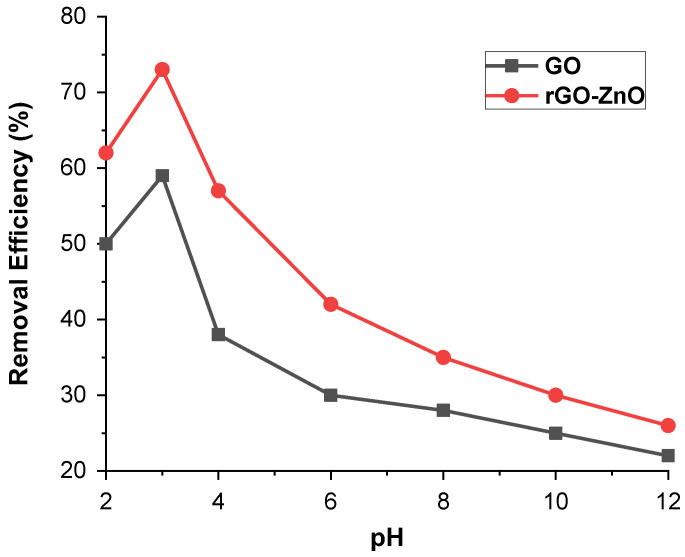
pH effect on % removal of Cr (VI) by synthesized adsorbents.

By maintaining other parameters as constant, such as 50 ppm adsorbate (Cr (VI)) and 60 mg adsorbent (GO and rGO-ZnO nanocomposite), the batch adsorption method was used to investigate the effect of a pH range of 2–12 on Cr (VI) ions uptake. Figure 6 displays the maximum removal of Cr (VI) at pH 3 and resulted in a decrease in removal efficiency if pH shift either to a higher or lower value. For GO, the maximum removal was 58% at pH 3 while for rGO-ZnO it was more than 72%. These results, therefore, show that acidic pH is a suitable medium for the removal of Cr (VI). It further reveals that in aqueous media, Cr (VI) ions may be present in the form of chromate (CrO_4_^−2^), dichromate (Cr_2_O_7_)^−2^, and hydrogen chromate (HCrO_4_^−^). Therefore, these three forms of Cr (VI) ions are associated with pH and overall chromate concentration. At a pH less than 6.8, HCrO_4_^−^ ions are present in greater amounts [24]. A pH greater than 6 shows no significant adsorption for Cr (VI).

#### 2.2.3. Effect of Temperature

**Figure 7:**
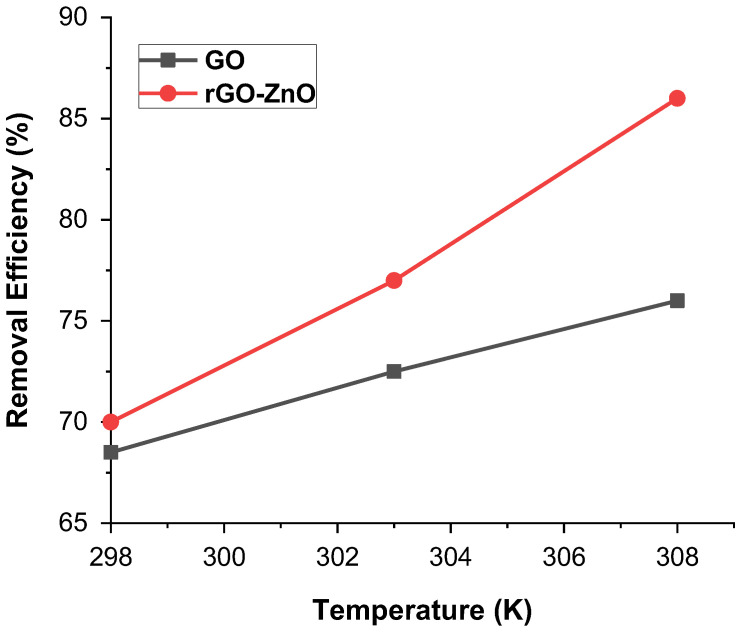
% Removal of Cr (VI) by adsorbents at various temperatures.

Temperature showed a pronounced effect on the sorption of Cr (VI). A minor change in temperature can have a greater effect on the interactive forces present in Cr (VI) and the adsorbent. This analysis was carried out at temperatures of 298, 303, and 308 K, while the sample was kept in the thermostat shaker. Results show that removal efficiency increases as the temperature increases. Figure 7 shows that rGO-ZnO nanocomposite shows higher adsorption of 85% at 308 K than that of GO, where 76% adsorption was achieved. In both cases, GO and rGO-ZnO nanocomposite, the percentage removal of Cr(VI) increases with temperature, as with a rise of temperature, adsorbate molecules obtain sufficient energy to reach active sites of adsorbent and establish enhanced interaction [32]. These results are in line with that deduced in Section 2.4, which also shows that adsorption is endothermic in nature.

#### 2.2.4. Effect of Contact Time

**Figure 8:**
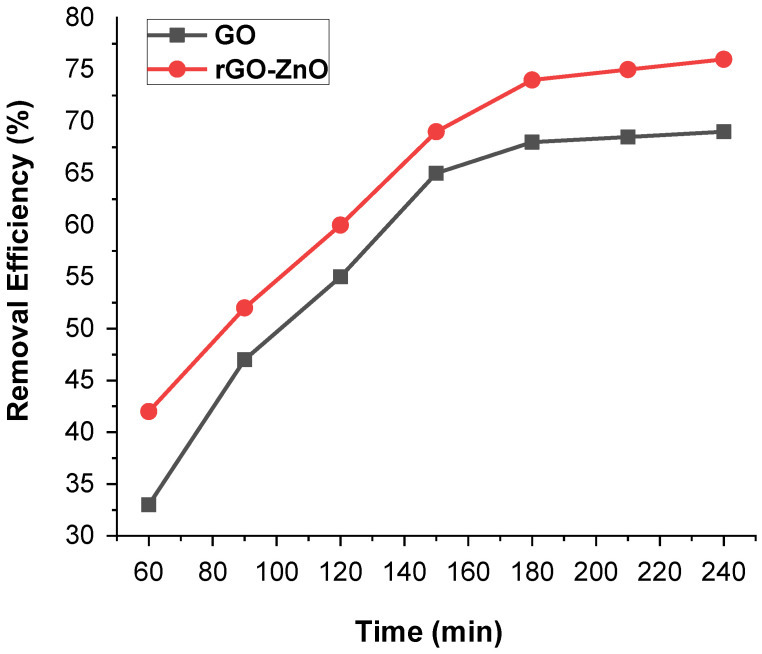
Contact time impact on the removal of Cr (VI) by GO and rGO-ZnO.

Time is a key factor for the economical water purification treatment process. The influence of contact time on the removal of the Cr (VI) was depicted in Figure 8. The time effect on the sorption capacity of GO and rGO-ZnO was promising when the initial concentration of Cr (VI) was 50 ppm (60 mg of adsorbent, pH = 3 and 298 K). It was observed that an increase in contact time from 1 h to 3 h promoted the removal efficiency. However, it remained constant even though the time was increased further. The maximum removal rates for GO and rGO-ZnO were estimated as 70% and 78%, respectively. These results indicate that rGO-ZnO is more efficient adsorbent than GO.

The initial increase in efficiency could be related to the availability of large vacant sites on adsorbent. After an initial gradual increase, a constant value was noted with the passage of time, showing that the vacant sites are well occupied.

#### 2.2.5. Effect of Adsorbent Mass

**Figure 9:**
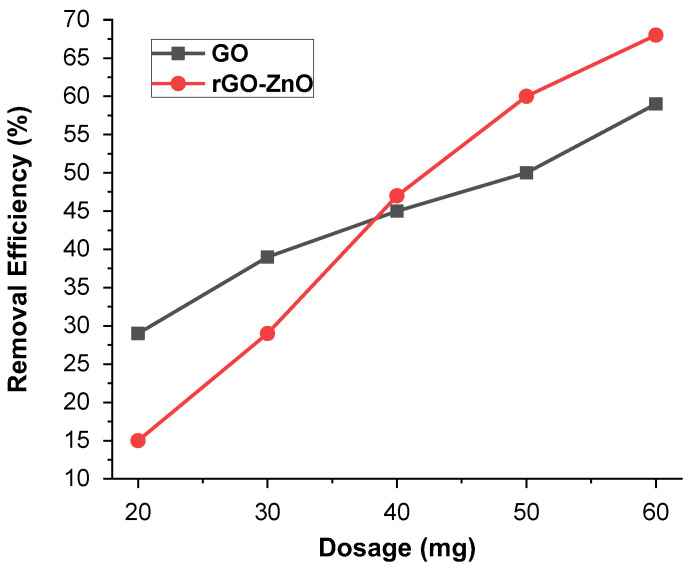
The effect of the mass on % removal of Cr (VI).

The impact of adsorbent mass (10–60 mg) for Cr (VI) was performed at pH 3 (Figure 9). It was observed that the ability to adsorb Cr (VI) vary by changing the adsorbent mass. An increased adsorbent mass with greater number of sites showed the maximum removal efficiency of 59% in the case of synthesized GO, whereas for rGO-ZnO, it was up to 68.6%. The higher adsorbent mass leads to greater removal of metal ions and is an important driving force to eliminate all mass transfer resistance of metal ions across aqueous/solid phases. Further increasing the adsorbent mass has no effect on the adsorbent’s removal efficiency, because it causes active sites to become closer together. The result showed that the rGO-ZnO nanocomposite is more efficient than the GO for Cr (VI) ion removal at high dosage levels.

### 2.3. Kinetic of Adsorption

A study of the adsorption kinetics of graphene oxide and rGO-ZnO was conducted using pseudo-first- and pseudo-second-order kinetic models. The kinetic data were found to be fitted with the following pseudo-first- and pseudo-second-order kinetic models Equations (2) and (3):

(2) $$log\left(q_{e-}q_{t\;}\right)=logq_{e\;\;}-\frac{k_{1\;}t}{2.303}$$

(3) $$\frac{t}{q_{t}}=\frac{1}{k_{2}q_{e^{2}}}+\frac{t}{q_{e}}$$

Adsorption capacity of sorbent was calculated by Equation (4):

(4) $$q_{e}=\frac{\left(C_{i}-C_{e}\right)V}{m}$$

In Equation (6), *q_e_* is the adsorption capacity and $C_{e\;}$ and $C_{i}$ are, respectively, the equilibrium and initial Cr (VI) concentrations (mg L^–1^) [3]. Equation (5) is the linear form of the Freundlich sorption isotherm, which reflects the dependence of extent of sorption on concentration:

(5) $$lnq_{e}=lnk_{f}+\frac{1}{n}lnC_{e}$$

where $K_{f}$ and *n* are Freundlich empirical constants signifying the sorption capacity and intensity, respectively. The greater the *n* is, the better the sorption ability and 1/*n*, which is the heterogeneity index, whose lower value reflects greater extent of heterogeneity [33].

The Langmuir model (Equation (6)), on the other hand, explains binding strengths between adsorbate and adsorbent:

(6) $$\frac{C_{e}}{q_{e}}=\left(\frac{1}{q_{m}b}\right)+\left(\frac{1}{q_{m}}\right)C_{e}$$

where $q_{m}$ (mg/g) and b are the maximum adsorption capacity and Langmuir constant, respectively [34].

**Figure 10:**
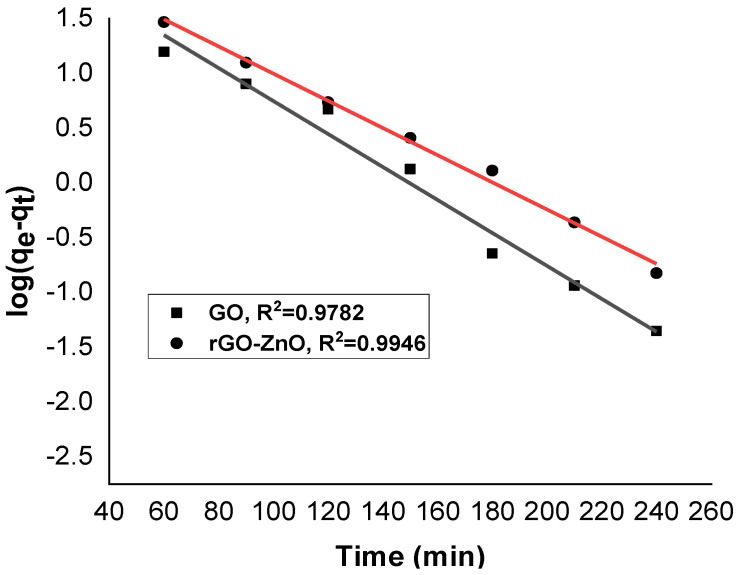
Pseudo-first-order kinetic model for Cr (VI) sorption.

**Figure 11:**
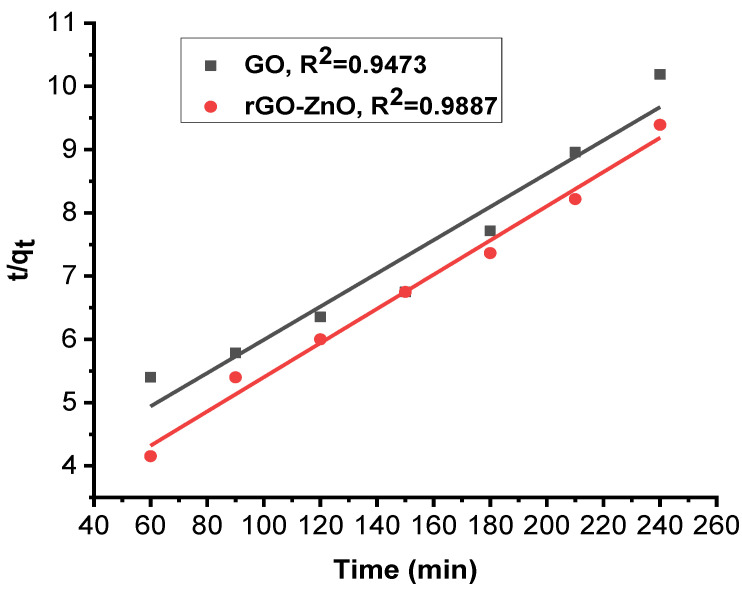
Pseudo-second-order kinetic model for Cr (VI) sorption.

Figure 10 and Figure 11 show linearized pseudo-first- and pseudo-second-order graphs for synthesized GO and rGO-ZnO, respectively. Based on the correlation coefficient, the pseudo-first-order kinetics (R² = 0.9946) fits better to sorption data by rGO-ZnO than GO. Similar results were reported elsewhere for the removal of Cr (VI) [35].

**Figure 12:**
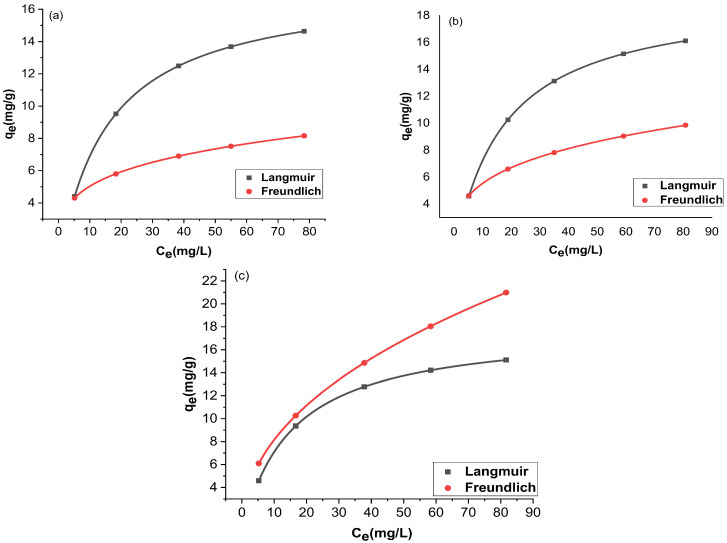
Nonlinear fitting of GO at 298 K (**a**), 303 K (**b**), and 308 K (**c**).

**Figure 13:**
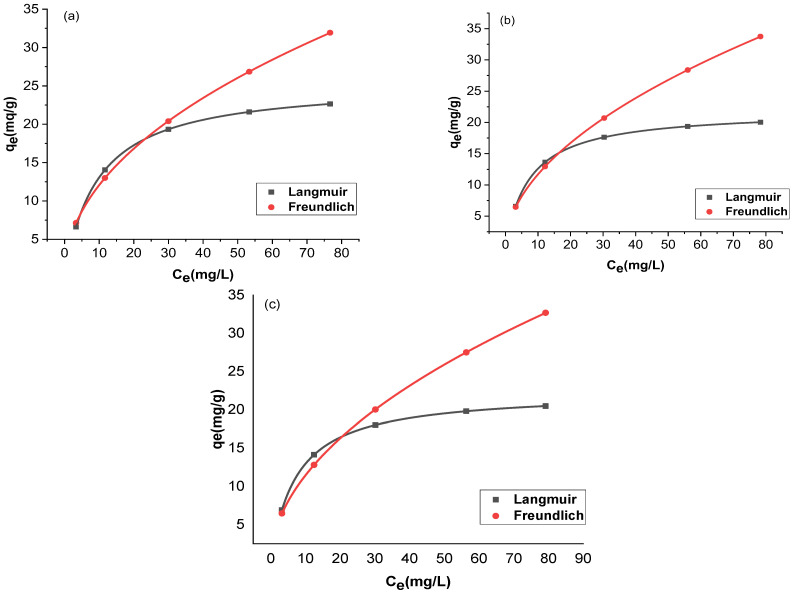
Nonlinear fitting of rGO-ZnO at 298 K (**a**), 303 K (**b**), and 308 K (**c**).

The adsorption and the residual adsorbate concentration were compared with two isotherms, Langmuir and Freundlich. The trial-and-error procedure was developed for non-linear regression to determine isotherm parameter factors/coefficients by maximizing determination. Figure 12 and Figure 13 show the non-linear forms of the Langmuir and Freundlich isotherms at three different temperatures. Table 1 provides a comparison of the isotherms for adsorption by Langmuir and Freundlich isotherms. The chromium adsorptions for GO and rGO-ZnO followed both Langmuir and Freundlich isotherms. The higher value of the correlation coefficient R^2^ supports this observation (Table 1). The capacity of the adsorbent to uptake chromium was found to be increased at higher temperatures.

In the literature, various materials/adsorbents are used for the effective removal of Cr (VI) from aqueous solution, such as crystalline hydrous titanium oxide [36], bentonite clay [37], calcined bauxite [38], fly ash [39], humic acid [40], and fuller’s earth [41]. Table 2 compares the adsorption capacities of GO and rGO-ZnO for removal of Cr (VI). According to this table, rGO-ZnO nanocomposite exhibited a high capacity for adsorption, whereas adsorbents such as fly ash, calcined bauxite, bentonite clay, and others exhibited a moderate capacity for adsorption, while TiO_2_ and biochar exhibited a negligible capacity for adsorption. We also observed that the adsorption capacity of activated alumina, as reported elsewhere, was comparable to that of the rGO-ZnO [42]. The adsorption capacity of crystalline hydrous titanium oxide was comparable with that of the synthesized GO nanosheets [43]. If we compare synthesized GO with rGO-ZnO nanocomposite, it is observed that rGO-ZnO recorded quite a higher adsorption capacity than GO.

### 2.4. Thermodynamic Studies

The thermodynamics of sorption infers whether the process is spontaneous or external energy driven. To better understand the effects of temperature on chromium removal, thermodynamic parameters were acquired using the Van ‘t Hoff equation:

(7) ΔG=ΔH−TΔS

(8) $$lnK=\frac{\Delta S}{R}-\frac{\Delta H}{RT}$$

where *T* denotes absolute temperature, *K* is the equilibrium constant, and R^2^ (8.314) is the general gas constant. To calculate the value of Δ*G*, Equation (7) was used. The slope and intercept of Van ‘t Hoff’s *lnK* vs. 1/*T* of Equation (8) are used to calculate the values of Δ*H* (change in enthalpy) and Δ*S* (change in entropy), respectively. If Δ*G* is a negative value, the fundamental criteria for the reaction occur spontaneously at a certain temperature.

The positive values of both ΔS and ΔH and the negative value ΔG reveal spontaneous and endothermic adsorption processes, as depicted in Table 3. The positive value of Δ*S* suggests some structural changes has been taken place at liquid solid interface. For the Langmuir isotherm, the value of Δ*H* is (GO = 8.136 and rGO-ZnO = 20.09), indicating the uptake of chromium on both GO and rGO-ZnO could be due to a physical as well as chemical adsorption process. Therefore, the sorption is not restricted to only monolayer formation.

### 2.5. Activation Parameter

Activation energy (*E_a_*) is a vital parameter and is calculated by linearized Arrhenius Equation (9):

(9) $$lnK=\ln A+\frac{E_{a}}{RT}$$

where *A* is the frequency factor and *K* is sorption rate constant. Slopes of the straight-line plot was used to calculate *E_a_* (Table 4). *E_a_* also indicates the nature of metal sorption. Due to the low energy requirement, equilibrium is usually achieved rapidly during physical adsorption and is easily reversible (*E_a_* = 0 to 40 kJ/mol) [38,45]. Chemical adsorption involves much stronger forces than physical adsorption, and therefore, activation energy varies with temperature (*E_a_* = 40 to 800 kJ/mol) [45]. For the chromium adsorption by GO and rGO-ZnO nanocomposite, the respective energy activation values were 8.14 and 20.09 kJ/mol. Values less than 40 indicate physical adsorption mechanisms [41]. Applying plants residues and NPs usage as adsorbents with sophisticated characterization methods have been successfully done earlier [28,46].

## 3. Materials and Methods

### 3.1. Reagents

In this study graphite, sulfuric acid (H_2_SO_4_ (98%)), potassium permanganate (KMnO₄), phosphoric acid (H_3_PO_4_; (85%)), potassium dichromate (K_2_Cr_2_O_7_), hydrogen peroxide (H_2_O_2_), hydrochloric acid (HCl), zinc nitrate hexahydrate (Zn(NO_3_)_2_.6H_2_O), and sodium borohydride (NaBH_4_) were used as purchased from Sigma Aldrich (Darmstadt, Germany). 

### 3.2. Synthesis of GO

For the synthesis of GO, a modified Hummer method was used [29,30,31]. In this technique, 3 mL of H_3_PO_4_ was reacted with H_2_SO_4_ (27 mL) in a 1:9 volume ratio. The stirring was performed for 30 min at 10 °C and then powdered graphite (0.225 g) was added, followed by the addition of KMnO_4_ (1.32 g) and the resultant mixture was agitated further for 6 h, leading to the formation of dark green solution. Then, 0.675 mL of H_2_O_2_ was slowly dropped to remove excess KMnO_4_. The entire reaction was performed in an ice bath, as it is highly exothermic in nature. After cooling it down, deionized water (30 mL) and HCl (10 mL) were added and centrifuged in micro centrifuge at 5000× *g* revolutions per minute (rpm) for 7 min. Finally, the GO powder was washed and dried at 90 °C for 24 h.

### 3.3. Synthesis of rGO-ZnO Nanocomposite

A cost-effective and single-step method was applied to prepare a nanocomposite of rGO-ZnO [25]. The reduction of GO and Zn (NO_3_)_2_ × 6H_2_O resulted in the formation of the nanocomposite of rGO-ZnO, which was prepared at room temperature. For the reduction, NaBH_4_ was used as a reducing agent. The GO (200 mg) was added in 200 mL of deionized (DI) water and sonicated until the appearance of a yellow suspension followed by addition of 2 g of Zn (NO_3_)_2_ × 6H_2_O at room temperature. It was sonicated (1 h) and then added to solution of 200 mL of 0.1 M NaBH_4_. The resulting viscous blackish-brown material was centrifuged, washed, and dried at 70 °C for 24 h. Appearance of light brown powder is an indicator for the formation of nanocomposite of rGO-ZnO.

### 3.4. Characterization

The X-ray diffractometer (XRD) is widely used for the detection of crystalline components as well as cell dimension analysis. The crystallinity of the synthesized rGO-ZnO nanocomposite was determined using PAN analytical X’Pert Pro by CAE, Montreal, Canada. Scanning electron microscopy (SEM) is a better imaging tool for particles, both at the nano and micro scales. In this work, SEM model Tescan Mira 3 was used to analyze the surface morphology of synthesized nanocomposite. The Fourier transform infrared spectroscopy (FT-IR) is frequently used to measure the presence of different characteristics in a given sample analysis. The KBr pellet method was used to investigate functional groups present in GO and rGO-ZnO by FT-IR-8400S-Shimadzu spectrophotometer (Japan) in the spectral range of 400–4000 cm^−1^ with a resolution of 4 cm^−^^1^. Ultra-violet and Visible (UV-Vis) is either a reflective spectroscopy or an absorption spectroscopy in a portion of the ultraviolet and in all adjacent noticeable spectral areas. A UV-Vis spectrophotometer (1601 SHIMADZU, Tokyo, Japan) was used to calculate wavelength maxima and band gap energy.

### 3.5. Adsorption Study

The batch adsorption method was used to conduct the sorption experiments. The stock solution (1000 mg/L) of potassium dichromate (2.826 g/L) was prepared from which different concentrations of chromium (10, 25, 50, 75, and 100 mg/L) were made by the dilution method. The optimum pH (3) of the chromium solution was achieved by using 0.1 M of each HCl and NaOH solution. In the adsorption experiments, 60 mg of each GO and rGO-ZnO nanocomposite were separately dispersed in each flask of 40 mL of chromium solution (10, 25, 50, 75, and 100 mg/L). The flasks were then transferred to a water shaker bath and agitated for four hours under various temperatures (298, 303, and 308 K). A UV-Visible spectra of the sample was recorded in order to estimate the Cr (VI) ion concentration in solution by using calibration curve. In order to study the effect of contact time, dosage, pH, and concentration on the adsorption of Cr (VI), different adsorption experiments were performed. Finally, isotherm and kinetics studies were applied to the acquired sorption data. The chromium solution removal efficiency was determined by Equation (10):

(10) $$\mathrm{Removal}\;\mathrm{Efficiency}=\left(\frac{C_{i}-C_{e}}{C_{i}}\right)\times 100$$

where $C_{e}$ and $C_{i}$ are chromium’s equilibrium and initial concentrations, respectively. The sorption capacity $q_{e}$ (mg/g) was calculated by Equation (11):

(11) $$q_{e}=\left(\frac{C_{i}-C_{e}}{m}\right)\times V$$

where m is the mass of adsorbent (g) and V is the volume of adsorbate solution (L).

## 4. Conclusions

This work presents a Cr (VI) removal by a simple and cost-effective adsorbent. The maximum Cr (VI) removal was achieved at pH 3. The highest uptake percentage for Cr (VI) ions was obtained within 180 min and increased adsorption rate was noted when the adsorbent mass was 0.5 g but further increase in mass was found ineffective. The Langmuir model and pseudo-first-order kinetics were suited well to sorption data. The removal performance of rGO-ZnO was better than GO under selected sorption parameters. The positive value of Δ*H* showed an endothermic nature while negative value of Δ*G* reflects favorable sorption. The values of Ea indicated that the nature of Cr (VI) sorption on both GO and rGO-ZnO was via physisorption. The overall sorption study, therefore, indicated that chemically prepared rGO-ZnO can be a better alternative to GO for efficient chromium uptake.

## Figures and Tables

**Table 1:** Non-linear fitting of models for Cr (VI) adsorption.

Sample	Temp. (K)	Langmuir Parameters			Freundlich Parameters		
		R^2^	*K_L_*	*q_m_* (mg/g)	R^2^	*K_f_*	1/*n*
GO	298	1	0.06	17.50	0.999	0.59	0.24
	303	1	0.06	19.49	0.999	0.59	0.28
	308	1	0.07	17.92	0.998	0.58	0.46
rGO-ZnO	298	1	1.06	25.45	0.999	0.40	0.48
	303	1	0.14	21.92	0.999	0.36	0.52
	308	1	0.14	22.37	0.998	0.36	0.51

**Table 2:** Sorption capacities of various reported adsorbents for Cr (VI).

Adsorbent	*q_m_* (mg/g)	References
Bentonite clay	0.572	[37]
Calcined bauxite	2.021	[38]
Fly ash	23.86	[39]
Humic acid	2.75	[40]
Fuller’s earth	23.58	[41]
Activated alumina	25.75	[42]
Crystalline hydrous titanium oxide	20.00	[40]
GO	1.222	[44]
GO	19.49	Present study
rGO-ZnO Nanocomposite	25.45	Present study

**Table 3:** Thermodynamic parameters for Cr (VI) sorption by GO and rGO-ZnO.

	Temp (K)	Δ*S* (kJ/(mol K))	△*H* (kJ/mol)	Δ*G* (kJ/mol)
GO	298	3.91	8.136	−2420
	303			−2461
	308			−2502
rGO-ZnO	298	49.02	20.086	−5936
	303			−6036
	308			−6137

**Table 4:** *E_a_* obtained from the linearized Arrhenius equation.

	Temp. (K)	1/*T*	*ln K*	*E_a_* (kJ/mol)
GO	298	3.3	−2.7	8.14
	303	3.3	−2.8	
	308	3.2	−2.7	
rGO-ZnO	298	3.3	−2.2	20.09
	303	3.3	−2.0	
	308	3.2	−1.9	

## Data Availability

All the data are enclosed in the manuscript.

## Sample Availability

The prepared samples are available from the authors, i.e., W.W. and T.N.
